# Making the case that episodic recollection is attributable to operations occurring at retrieval rather than to content stored in a dedicated subsystem of long-term memory

**DOI:** 10.3389/fnbeh.2013.00003

**Published:** 2013-02-01

**Authors:** Stanley B. Klein

**Affiliations:** Department of Psychological and Brain Sciences, University of CaliforniaSanta Barbara, CA, USA

**Keywords:** episodic memory, semantic memory, autonoetic awarness, amnesia, self, consciousness

## Abstract

Episodic memory often is conceptualized as a uniquely human system of long-term memory that makes available knowledge accompanied by the temporal and spatial context in which that knowledge was acquired. Retrieval from episodic memory entails a form of first–person subjectivity called autonoetic consciousness that provides a sense that a recollection was something that took place in the experiencer's personal past. In this paper I expand on this definition of episodic memory. Specifically, I suggest that (1) the core features assumed unique to episodic memory are shared by semantic memory, (2) episodic memory cannot be fully understood unless one appreciates that episodic recollection requires the coordinated function of a number of distinct, yet interacting, “enabling” systems. Although these systems—ownership, self, subjective temporality, and agency—are not traditionally viewed as memorial in nature, each is necessary for episodic recollection and jointly they may be sufficient, and (3) the type of subjective awareness provided by episodic recollection (autonoetic) is relational rather than intrinsic—i.e., it can be lost in certain patient populations, thus rendering episodic memory content indistinguishable from the content of semantic long-term memory.

What is episodic memory? As initially conceptualized, it is a system of long-term memory whose content provided its owner with a record of the temporal, spatial and self-referential features of the context in which the original learning transpired (e.g., Tulving, 1972, 1983). By contrast, semantic long-term memory lacked these features: Its offerings were experienced as knowledge devoid of the contextual elements in which it was acquired.

A clear implication of this distinction was the difference in subjective relations these systems of memory shared with the past. Episodic memory entailed awareness that a current recollection referred directly to, and thus was conceptualized as, an event that had transpired previously in one's life. By contrast, content from semantic memory was solely occurrent—it was present to awareness—as either thought or image—and though one could logically infer that the occurrent content must have been acquired at some time in one's past, the recollection of its acquisition was not part of its experienced presentation.

These temporal distinctions were fully appreciated by Tulving, and in 1985 he made them an explicit basis for distinguishing between episodic and semantic systems of memory (Tulving, 1985). Adopting terminology proposed originally by Husserl—“noesis” (i.e., the type of experience associated with thought and remembering; e.g., Husserl, 1964)—Tulving focused attention on the types temporal subjectivity accompanying the retrieval of episodic and semantic memory. Episodic memory was held to enable *autonoetic* awareness, while semantic memory enabled a type of awareness he termed *noetic* (e.g., Tulving, 1985, 1993, 2002; Wheeler et al., 1997; Szpunar and Tulving, 2011).

Autonoetic awareness was defined as “self-knowing”: when autonoetically aware, the individual's phenomenology is characterized by “… a unique awareness of re-experiencing here and now something that happened before, at another time and in another place” (Tulving, 1993, p. 68). By contrast, noetic awareness occurs when one thinks objectively about something one knows. Individuals are said to be noetically aware when “they retrieve general information in the absence of a feeling of re-experiencing the past” (Szpunar, 2010, p. 144). Central to the proposed distinction: “Only ‘autonoetic consciousness’ is thought to bear a personally meaningful relation to time” (Szpunar and Tulving, 2011, p. 4).

Autnoetic and noetic awareness align naturally with episodic and semantic modes of remembering, respectively (Tulving, 1985, 1993, 2002; Wheeler et al., 1997). Only autonoetic experience is assumed capable of providing the subjective requirements for mental time travel (e.g., Suddendorf and Corballis, 1997, 2007; Wheeler et al., 1997; Tulving, 2002; but see Klein, 2013b). Accordingly, episodic memory is tied directly to temporally-rich autonoetic experience. By contrast, awareness of semantic knowledge (i.e., noetic) lacks a subjective sense that one is mentally traveling to back in time to the events and experiences in one's past that gave birth to that awareness.

Tulving's reformulation of episodic and semantic memory in terms of temporal subjectivity has been widely adopted by memory researchers and has shown to be a particularly fruitful way of generating testable hypotheses and theoretical models of the episodic and semantic division of long-term declarative memory (e.g., Perner and Ruffman, 1994; Gardiner, 2001; Piolino et al., 2003; Wheeler, 2005; Markowitsch and Staniliou, 2011; for reviews, see Wheeler et al., 1997; Dere et al., 2008). A distinction based on subjective temporality also avoids a number of untidy findings that, over the years, have chipped away at the traditional classification of episodic and semantic memory in terms of the presence or absence of the criteria of time, space and self.

For instance, the assumption that episodic, rather than semantic, memory entails a self-referential component has given way to the well-recognized fact that knowledge in semantic memory also can be self-referential (for reviews, see Klein, 2010; Klein and Gangi, 2010; Klein and Lax, 2010; Renoult et al., in press). In addition, the content of semantic memory is capable of contributing to a knowledge-based representation that includes both spatial and temporal contextual information (e.g., “I know that John Lennon was born on October 9th, 1940 in Liverpool, UK, although I do not recollect the event in which that knowledge was acquired”; e.g., Tulving et al., 1988; Kopelman et al., 1989; Klein, 2001; for recent reviews see Klein, 2004; Klein and Gangi, 2010; Klein and Lax, 2010; Martinelli et al., 2012).

Thus, the core constituents of episodic memory as initially proposed (i.e., temporal, spatial, and self-referential) also can be on display in semantic memory experience. Indeed, there appears no principled reason why the content of these two systems should differ. This is demonstrated rather dramatically in a case presented by Stuss and Guzman (1988). An individual, who, as a result of illness, suffered profound retrograde episodic amnesia, nonetheless was able to successfully re-learn specific temporal and spatial details of his personal past. However, in accord with the autonoetic/noetic distinction, the patient experienced this content as semantic fact rather than as a re-living of his personal past (since he did not suffer comparable anterograde impairment, he could, of course, mentally travel back to the experienced events in which personal knowledge was relearned).

Thus, there is no logical argument or empirical evidence supporting the assertion that only episodic memory makes reference to the “where and when” of past personal experience. While the fact that two potentially separate systems share criteria is not a “death sentence” for a taxonomy, it highlights the severe difficulties faced by those who would adopt the “time, place, and self” criteria as *the* basis for classification.

In summary, the “time, space, and self” criterion for distinguishing between semantic and episodic memory is insufficient for the task for which it originally was designed. By contrast, the autonoetic/noetic criterion for classification does good work both in capturing a fundamental feature of our memory phenomenology and in serving as fertile theoretical and empirical grounds for exploring the complexities of the types of systems subsumed under the general designator “memory” (for reviews, see Wheeler, 2000; Markowitsch, 2003; Dere et al., 2008). In the next section I discuss issues involved in identifying an occurrent mental state as a memory.

## Memory experience and its connection to the past

The adoption of subjective temporality as a basis for distinguishing between forms of memory trades on the notion that the criteria for classification lie not in the *form* of content experienced, but in the *manner* in which that content is received by consciousness. In one sense, all memory-based content is experienced as occurrent—it is an act of mind happening *now*. As Reid puts it, “Every man can distinguish the thing remembered from the remembrance of it. We may remember anything we have seen, or heard, or known, of done, or suffered; but the remembrance of it is a particular act of the mind which now exists, and of which we are conscious” (Reid, 1813/1969, pp 324–325). It is the attachment of the “past” to present mental experience (be it imagistic or propositional) that marks the experience as one of memory rather than, say, imagination (Reid, by the way, denied the possibility of imagistic recollection, but that need not concern us at present).

The requirement that a current mental state evoke a sense of attachment to one's past to establish it as an act of memory (rather than, say, thought or imagination) long has been recognized. And, it has been a persistent thorn in the side of psychologists and philosophers grappling with the problem of placing a current mental state in a relation to the past. And, for the relation to do good work, it must be of the “right type.”

So, what is the “right type” of relation between an occurrent mental state and the past? William James (1890), as so often is the case, puts the problem in sharp relief: “A farther condition is required before the present image can be held to stand for a *past original*. That condition is the fact that the imagined be *expressly referred to the past*, thought as *in the past* … But even that would not be memory. Memory requires more than mere dating of a fact in the past. It must be dated in *my* past. In other words, I must think that I directly experienced its occurrence. It must have … ‘warmth and intimacy’… ” (James, 1890, p. 650; emphasis in original).

Over the years (both prior to and following James' remarks) numerous criteria have been proposed to do the work of differentiating memory from a non-memorial mental content.^1^ Hume famously proposed that the vivacity of a mental image is a basis on which we separate an image or thought from a memory, with memory being more lively and vivacious. He also proposed that the amount of “free play” we have with our mental states serves as a useful criterion—in contrast to imagining, for example, when we remember we have less free play with mental content, since that content is bound by the past to represent things as they actually were (Hume, 1748/2004). Russell saw things differently, proposing that to be considered a memory a mental content must be accompanied by two feelings—pastness and familiarity (Russell, 1921).

Among modern psychological investigators, the work of Johnson and her colleagues represents the most systematic attempt to tackle this vexing problem (for review, see Johnson and Raye, 1981; Johnson et al., 1993). Their research eventuated in a set of criteria—e.g., the richness of the contextual and perceptual elements contained in a mental state. Although these criteria were proposed primarily to account for discrimination between memories for thoughts and memories for perceptions, they easily are adapted for classifying mental content as a memory as opposed to an act of thought or imagination.

Unfortunately, as theorists and philosophers have discovered, none of these criteria stand the test of logical analysis or introspection (for reviews, see Furlong, 1951; Casey, 1977; Warnock, 1987; Bernecker, 2010). For example, Russell's and Hume's assumption that the content of memory experience is “bound to the past” is partly undermined by demonstrations that memorial experience is, at least to a degree, reconstructive (e.g., Bartlett, 1932). And, we all have had experiences in which an imagination is vivid and a memory faint (e.g., Warnock, 1987). As Bernecker (2010) concluded, the problem with the memory-markers thus far proposed is that “they don't offer a reliable mark” (p. 22). Each is subject to logical argument and/or empirical demonstration that makes clear that none of the proposed criteria are either necessary or sufficient for marking a memory as such.

Considered in this light, the autonoetic/noetic criterion seems encouraging. While it may not serve as a definitive basis for distinguishing semantic memory from imagination—both are, by definition, noetic—it does provide a promising conceptual and phenomenological basis for identifying present mental content as part of one's past. It thus appears to be up to the task—that is, it provides the “right type” of relation—when the issue at hand is to determine whether an occurrent mental content is an episodic memory.

## Is autonoetic awareness intrinsic to episodic recollection?

Having argued in support of Tulving's contention that temporal subjectivity can serve as a useful basis by which to classify mental content as memorial, the issue now at hand is this—does subjective temporality provide sufficient license to conclude that current mental content represents an episodic, as opposed to a semantic, memory? As noted, content alone is not sufficient to make this judgment, since the constituents of both systems of memory *can* include temporal, spatial and self-referential elements. By contrast, the mode of subjective temporality accompanying an act of memory is assumed to differ dramatically between episodic recollection and semantic retrieval.

The content retrieved from semantic memory, on Tulving's account, can (at most) be *about* the past. Retrieval from semantic memory can be taken as either temporal (Klein et al., 2002b; for review, see Klein, 2013a) or atemporal (e.g., Klein et al., 2010). What determines its stance with respect to time is not the *quality* of the experience *per se*, but rather our ability to inferentially *refer* the experience to the past on the basis of the content present in (noetic) awareness. Thus, a causal analysis is required to place an occurrent semantic memory in a relation to the past.

On the other hand, episodic memory's connection to the past is not one of logical inference. Rather, it is pre-reflective, directly *given* (e.g., Zahavi, 2005). It is a sense (i.e., a feeling) that my current mental state stands for, and thus representative of, an experience in my personal past (e.g., James, 1890). The content of episodic recollection is given as being *of* the past; it is accompanied by a feeling of mental time travel—that is, re-visiting a personal experience. This feeling, or sense of attachment, to my past (which James called “warmth and intimacy” and Russell labeled “feelings of familiarity and pastness”), is part of the subjective *quality of* the mental event. As Nagel (1974) might say, the pastness of an episodic experience is part of “the feeling of what it is like” to have such an experience. And this feeling (or qualia) accompanying episodic recollection is made possible by episodic memory's association with autonoetic awareness.

The distinction between episodic and semantic memorial experience can thus be seen as one of differences in manner of acquaintance (e.g., Russell, 1912/1999). We are acquainted with semantic pastness indirectly via inference, whereas our acquaintance with episodic pastness is directly given as the feeling that we are re-living our past. If this distinction holds, then a phenomenological state is what differentiates our experience of these two forms of long-term memory.

So what exactly is the *connection* between autonoetic awareness and episodic memory? Two possibilities present themselves. Either (as commonly assumed, though seldom stated), autonoetic awareness is (1) *intrinsic* (i.e., necessary) to episodic memory—i.e., it is a part, or constituent, of “episodic” content, or (2) it has a *relational* (i.e., contingent) connection to memory content—i.e., while under normal circumstances it is observed to be coextensive with “episodic” content, this connection is one of contingency rather than necessity.

On the relational view, the neuro-cognitive mechanisms that make possible autonoetic awareness are functionally independent of the mechanisms that make available the content of long-term memory. What makes a memory experience episodic or semantic is not the nature of the content, or the hypothesized system in which content resides while in “storage,” but rather an act of temporal (or atemporal) awareness that becomes associated with the content *once* it has been retrieved.

This is the view I champion in this paper. Although evidence in support is scarce, suggestive findings have been reported in a study by Piolino and colleagues (2003). Using the remember/know paradigm (e.g., Tulving, 1985), these investigators found that patients suffering Alzhiemer's Disease and Frontotemporal Dementia report significantly fewer “remember” responses than do controls. Based on this finding, they conclude that these two forms of dementia entail impairments specific to autonoetic awareness. However, whether this impairment occurs in storage or at retrieval is indeterminate. Moreover, as I discuss in section “Conclusions,” the proposed relation between remember judgments and autonoeisis is more suggestive than theoretically grounded. I present evidence that bears directly on the relation between autonoetic awareness and retrieval in section “Can Autonoetic Awareness be Separated from Episodic Content? The Case of Patient R. B.”

In sum, the relational view implies that memory content is neither episodic nor semantic; rather retrieved content is classifiable as episodic or semantic by virtue of a concurrent act of autoneotic awareness, whose association (or lack thereof) is used to classify an occurrent mental state as episodic or semantic. Seen in this light, there are no systems of episodic and semantic memory *per se*. Rather, there is memory content that can, if a suitable candidate for temporal specification is present in consciousness (e.g., “where and when I learned that 2 + 2 = 4,” but not “I know that 2 + 2 = 4”) can be acted on by the autonoetic subsystem to confer a sense of temporal subjectivity on the content retrieved.^2^

## Episodic memory: a componential approach

Many contemporary treatments of episodic memory focus on the encoding, storage and retrieval of memory content (for discussion, see Klein, 2007). However, as Klein et al. (2004) have argued, theoretical and empirical considerations call into question the wisdom of such restrictive treatment of memory experience. Their work makes a strong case that episodic recollection entails a multiplicity of functionally independent, yet normally interacting, systems, only some of which bear an obvious a priori relation to memory taken as the encoding, storage and retrieval of content.

Specifically, Klein et al. (2004) proposed that to experience memory content as episodic requires, at a minimum, four capabilities. These include (1) a capacity for self-reflection; that is, the ability to reflect on my own mental states—to know about my own knowing, (2) a sense of personal agency; that is, the belief that I am the cause of my thoughts and actions, (3) a sense of personal ownership; that is, the feeling that my thoughts and acts belong to me, and (4) the ability to think about time as an unfolding of personal happenings centered about the self. Klein and colleagues conceptualized episodic recollection as a mental state resulting from the finely tuned interplay of these four psychological capacities that, working together, transform a retrieved content into an episodic experience. It follows that breakdowns in any of these components (self-reflection, self-agency, self-ownership, and personal temporality) should produce, in varying degrees, specific, highly circumscribed impairments in episodic recollection. A review of the available evidence showed that this does indeed occur (e.g., Klein, 2001; Klein et al., 2004), leading the authors to suggest that the subsystems identified may provide a set of individually necessary and jointly sufficient conditions for enabling memory content to be experienced as episodic recollection.

I will not review the specifics of the subsystems identified by Klein et al. (2004; for a related view, see Klein, 2011). Rather, in what follows I focus on one system with direct relevance to present concerns—the system that enables a feeling of ownership of one's mental states—and I discuss its implications for autonoetic awareness and its relation to episodic memory experience.

## Can autonoetic awareness be separated from episodic content? the case of patient R. B.

As noted in Section “Is Autonoetic Awareness Intrinsic to Episodic Recollection?” if autonoetic awareness is functionally independent from memory content, then temporal subjectivity and memory content should be capable of being pried apart. The case of patient R. B. (reported below), has direct bearing on this issue.

The idea that ownership of one's mental states can come loose from the states, as experienced, is not a novel observation: A substantial literature (primarily clinical) speaks to the reality of this possibility. For example, in a largely speculative treatment, Jaynes (1976) argued that prior to acquiring the capacity to confer ownership on conscious thought our ancestors were unable to accurately localize the origination of “voices heard” in their heads. Less speculative evidence comes from cases of thought insertion in schizophrenics suffering delusional symptoms (e.g., Frith, 1992; Gallagher, 2000; Northoff, 2000; Bortolotti and Broome, 2009; for review, see Stephens and Graham, 2000). However, while these cases support the realizability, and thus conceivability, of a lack of personal ownership of one's mental states, schizophrenic patients *apparent* memories, even in early stages of the disorder, likely are delusions in some sense (e.g., Northoff, 2000; Klein and Nichols, 2012), and this renders such cases less than optimal as demonstrations that memory (as opposed to, say, delusion) and ownership can come apart.

Of particular importance, then, is the case of patient R. B. (the details are summarized herein. A fuller treatment can be found in Klein and Nichols, 2012). As a result of a serious accident, R. B. suffered, in addition to severe physical injuries, a number of cognitive impairments. These included difficulty in maintaining attention, mild aphasia, and retrograde and anterograde amnesia for events in close temporal proximity to the accident. His performance on tests of verbal fluency and short-term memory span fell below the scores provided by neurologically healthy age-matched controls.

While in the hospital, R. B. was placed on morphine (IV drip, followed by pills) and Oxycontin to alleviate the considerable pain he endured. As the intensity of his pain subsided, he weaned himself off medication. Importantly, at the time of testing R. B. was not on any pain medication. In addition, his long and short-term memory impairments, aphasia and fluency deficits had resolved.

Not all cognitive function, however, had returned to normal. Of direct bearing on the question at hand—the relation of autonoetic awareness to episodic memory—R. B. was able to remember particular incidents from his life accompanied by clear temporal, spatial and self-referential content. But he did not *feel* the content experienced belonged to him. In his words, they lacked “ownership”^3^ (in the descriptive language of James, Russell, and Hume, his “memories” lacked feelings of warmth, intimacy, and personal pastness). Viewed in terms of episodic criteria, his mental states presented content adhering to the original episodic criteria, but divorced from autonoetic awareness.

This particular type of memory impairment—recollection absent a sense of personal ownership—is a form of memory dissociation that, to my knowledge, has not previously been documented in the amnesic literature (Klein and Nichols, 2012). There are, however, two cases that bear similarity. One is from a study of an amnesic reported by Talland (1964). Unfortunately, the data available from that brief report, while suggesting a similar dissociation, are too limited to support strong conclusions. In the other case (touched on earlier in this paper), a patient relearned his “personal history” following a case of severe retrograde episodic amnesia spanning most of his past life (Stuss and Guzman, 1988). The patient commented that the relearned memories seemed to lack a feel of real happenings in his life. They were, to him, more like stories and facts told to him by others (which, indeed, they were!). In this sense, they were more like semantic facts about himself (e.g. Klein and Gangi, 2010; Klein and Lax, 2010) than episodic recollections: The patient knew his memories were about him, but he did not remember them as temporally and spatially acquired in the correct context (that is, when the original acts transpired). Instead, they were memories that temporally and spatially were correctly experienced as second-hand stories told to him at a particular time and place (i.e., they consisted in the recollection of information acquired following the onset of his retrograde memory loss).

So, are R. B.'s memories episodic or semantic? The reported content suggests the former, but R. B.'s reported experience suggests the latter. Almost immediately following his accident, R. B. was able to intentionally retrieve specific events temporally and spatially situated in his personal past. But, as noted, his memories were compromised in an unusual manner—retrieval of events, though fitting the standard criteria for episodic recollection (i.e., time, place, and about R. B.), were unaccompanied by a sense of personal ownership. And, absent that sense, the ability to *feel* these memories as emanating from his personal past—to mentally travel to the time in which the events represented by his current thought and images originally transpired—was lost.

Lacking the experience of personal ownership, R. B. simultaneously lost a direct, personal connection with his past. It thus appears that loss of ownership equates to an inability to draw on the resources provided by autonoetic awareness to identify the content of a current mental state as a part of one's personal history. For example, approximately 2 months following release from the hospital, R. B. offered the following description of what it is like for him to recall personal events:

“… I did not own any memories that came before my injury. I knew things that came before my injury. In fact, it seemed that my memory was just fine for things that happened going back years in the past (the period close to the injury was more disrupted). I could answer any question about where I lived at different times in my life, who my friends were, where I went to school, activities I enjoyed. But none of it was ‘me.’ It was the same sort of knowledge I might have about how my parents met or the history of the Civil War or something like that.”

In my review of taxonomies of long-term memory, I noted that psychologists traditionally have characterized episodic recollections as temporal, spatial, and self-referential. By these criteria, R. B.'s descriptions of his memorial experience leave little doubt that they are episodic recollections, appropriately situated in time and space, rather than factual, atemporal semantic knowledge. As I also suggested, however, there is no principled reason why a semantic fact could not contain spatial, temporal and self-referential content, or be correctly referred to a person's past—albeit a past constructed inferentially rather than autonoetically given. And therein resides a puzzle. Do R. B.'s unowned memories consist in episodic content divorced from its relational connection to autonoetic awareness, or are they rather un-categorized (i.e., as episodic or semantic) content retrieved from storage but divorced from the sense of personal ownership conferred by an act of autoneotic awareness?

R. B. addresses the question directly. When asked to recall of events from his childhood he replies:

“I was remembering scenes, not facts … I was recalling scenes … that is … I could clearly recall a scene of me at the beach in New London with my family as a child. But the feeling was that the scene was not my memory. As if I was looking at a photo of someone else's vacation.”

All of R. B.'s memories were substantiated by third parties as valid renditions of events that actually transpired in his life. While his recollections satisfy the traditional criteria for episodic memory—time, place, and self-accompanied by clear imagistic representation of (typically) unique events (see below for additional examples)—they simultaneously exhibit an absence of experienced ownership: While he can infer that the events recalled *must* be representative of past personal experience, he does not know this by virtue of a direct, felt connection to the past. In short, he has no sense of re-living the experiences retrieved.

The absence of ownership also is evident in R. B.'s response to instructions to recall personal memories from time spent in graduate school:

“I can picture the scene perfectly clearly … studying with my friends in our study lounge. I can ‘relive’ it in the sense of re-running the experience of being there. But it has the feeling of imagining, (as if) re-running an experience that my parents described from their college days. It did not feel like it was something that really had been a part of my life. Intellectually I suppose I never doubted that it was a part of my life. Perhaps because there was such continuity of memories that fit a pattern that lead up to the present time. But that in itself did not help change the feeling of ownership.”

He continues:

“Things that were in the present, like my name, I continue to own. Having been to MIT had two different issues … my memories of having been at MIT I did not own. Those scenes of being at MIT were vivid, but they were not mine. But I owned ‘the fact that I had a degree from MIT’… that might have simply been a matter of rational acceptance of fact.”

Once again, R. B.'s memory performance adheres to the traditional definition of episodic recollection: He can vividly remember where the specific event transpired, when the specific event took place, and that it directly involved him. But, autonoetic awareness is missing. In short, on the view presented here, the notions of episodic and semantic can be seen to refer not to different stores or systems of memory content, but rather to how that content, once retrieved, is acted on by mechanisms (inferential or autonoetic) to enact a reference to one's past (note: not all memory content can be mapped to the past^2^). This proposal is reflected in the way R. B. treats his perspectival, but not personally, owned pre-injury memory content, both during its separation from autonoetic awareness and following their eventual recoupling—at which point memory retrieval previously experienced as “unowned” takes on a decidedly episodic flavor:

SBK: “Can you recall personally important events from your pre-injury period?”

RB: “I remember things that came before my injury. In fact, it seems that my memory is just fine for things that happened going back years in the past. I can answer questions about where I lived at different times in my life, who my friends were, where I went to school, activities I enjoyed … but … to clarify … I am remembering scenes, not facts. Since I am remembering scenes, I think this means I am dealing with exactly what you are asking about.”

SBK: “Can you recall who you are? More specifically, what you were like and what you are like—that is, your trait characteristics. If so, are your traits felt as your own?”

RB: “Yes, I know what I am like … intelligent, shy, honest, a good person, things like that? Yes, I definitely have no identity problem. And the memories created since the injury I have full ownership of.”^4^

SBK: “Can you recall for me a personal event concerning your time at college that would involve knowing what happened to you as a personal experience. Or is the recall more of a factual nature?”

RB: “I can see the scene in my head. I'm studying with friends in the lounge at my residence hall. I am able to re-live it. I have a feeling … a sense of being at there, at MIT, in the lounge. But it doesn't feel like I own it. It's like I'm imagining, re-living the experience but it was described by someone else.”

SBK: “Can you recall memories whenever you desire to do so?”

RB: “I can recall memories (from the non-ownership period of his life) at will. I have normal control over remembering facts and scenes from my past. But when I remember scenes from before the injury, they do not feel as if they happened to me—though intellectually I know they did—they feel as if they happened to someone else.”

With respect to the recovery of episodic ownership:

“When I did ‘take ownership’ of a memory, it was actually quite isolated. A single memory I might own, yet another memory connected to it I would not own. It was a startling experience to have no rhyme or reason to which memories I slowly took ownership of, one at a time at random over a period of weeks and months.”

He continues:

“What happened over the coming months … was interesting. Every once in a while, I would suddenly think about something in my past and I would ‘own’ it. That was indeed something ‘I’ had done and experienced. Over time, one by one, I would come to ‘own’ different memories. Eventually, after perhaps 8 months or so, it seemed as if it was all owned … as if once enough individual memories were owned, it was all owned. For example, the MIT memory, the one in the lounge … I now own it. It's clearly part of my life, my past.”

### Summing up: the case of patient R. B. and its implications for episodic memory

There appears to be an intimate connection with, and possibility of separation between, the content of a memory experience and whether that experience is autonoetic. R. B.'s confusion with regard to “content ownership” highlights the intimate relation between autonoetic awareness and ownership: Absent a sense of ownership, R. B. cannot mentally travel back in time to claim as his own the re-presentation of the experience from which the occurrent content was derived. Instead he relies on inference and logical possibility to (often correctly) infer that, given the specific elements of the content retrieved, it likely represents aspects of his personal past.

The apparent paradox presented by the case of patient R. B.—episodic-like content absent the feeling of personal ownership (and thus lived pastness)—can be understood by situating episodic memory in the context of a system of interrelated memory processes, some of which provide the raw data for experience (i.e., content) and some of which enable the experience to be “mine” (for extended treatments, see Klein, 2004, 2010, 2012; Klein et al., 2004; Klein and Nichols, 2012). R. B.'s recollections during his “unowned” period can be explained in the context of the view that there is specialized neural machinery that acts on retrieved content (of the right sort; e.g., Footnote 2) to confer on it a *sense* of re-living a personal experience—i.e., episodic recollection. This neural machinery in R. B. seems to have been compromised by his injury, but only for those events that occurred in the time period preceding his injury. That is, R. B. suffered a form of retrograde amnesia that compromised his ability to experience his personal recollections as his own. During the non-ownership period, R. B. had memory of pre-injury events and could locate them, via inference, in his personal past. But he lacked a sense of numerical personal identity with the experiences retrieved. The case of R. B. thus suggests that the sense of numerical personal identity is quite narrowly circumscribed: R. B. had factual self-knowledge, trait self-knowledge, and knowledge of personally experienced episodes, but he did not have a pre-reflectively given sense of continuity with his past person.

Importantly, during his “unowned” period R. B. had no trouble retrieving very specific, often one-of-a kind, personal experiences (e.g., being on a beach in New London). He presumably also had no trouble representing that his body was present for those experiences. He *knew* that the memories were about him rather than, say, his mother. And he could call up, that is, auto-cue (Donald, 1991) memories at will. So, in that sense, his memories were both agentic and involved self-reference. However, there seems to be another type of self-reference that typically accompanies episodic recollections (ownership, mineness; for discussion, see Klein, 2012) that has been impaired in his case. His apparent deficit was in representing, from the first person, “I had these experiences.” That is, his impairment entailed a loss of the ability to connect personally experienced content with autonoetic awareness.

## A mechanism: one proposal

Although the specifics of R. B.'s deficit are, at this point, unclear, it is worth considering the present findings in light of theoretical work by Dalla Barba and colleagues (e.g., Dalla Barba, 2002; Dalla Barba et al., 1997, 1999) on the relation between consciousness, memory, and temporal experience. These authors call attention to two modes of consciousness, which they term temporal consciousness (TC) and knowing consciousness (KC). TC is consciousness of time—it enables a person to become aware of something as part of his or her personal past, present, or future. It thus corresponds closely to what Tulving calls autonoetic awareness. KC, by contrast, does not locate objects in time. Rather, it enables a person to become aware of something as an element of knowledge without that knowledge being situated in a temporal framework. The conceptual overlap with noetic awareness is evident.

TC and KC thus conceptualized are two different ways of *addressing* the contents of memory. Long-term memory is held to contain representations that vary in terms of their stability and resistance to modification (e.g., Dalla Barba et al., 1997, 1999; Nadel and Moscovitch, 1997). The more stable, overlearned, summary representations can be thought of as roughly analogous to what *will be* experienced, on retrieval, as semantic knowledge, whereas less stable, more malleable representations—e.g., unique, one-of-a-kind-events—provide the raw material for what will subsequently become episodic recollection.

Support for these proposed distinctions in content stability can be found in Nadel and Moscovitch's (1997; Moscovitch et al., 2005) multiple trace theory (MTT) of long-term memory consolidation (see also Piolino et al., 2003). According to MTT, the hippocampal formation and related structures (primarily in the medial temporal lobes) contribute to the transformation of initially unstable and sparsely encoded content into a collection of contextually related traces—a transformative act that confers stability on represented content by virtue of its multiple instantiations. The model, developed to map the neural events and structures underlying the transition of information from episodic to semantic memory (i.e., unstable to stable), fits reasonably well with Dalla Barba's proposal that differences in representational stability determine whether a given memory content will be taken an as object by TC and KC.

But, in what sense does the relative stability (or a lack thereof) confer a temporal status on a specific content? That is, what determines if the content, as experienced, is classified as episodic or as semantic? Dalla Barba (2002) suggests that the stability of a representation is correlated with what we typically describe as episodic memory by virtue of the fact that TC takes such content as its intentional object (for a discussion of why this may be the case, see Klein et al., 2002a). In this way, temporally and representationally unique events are likely to be experienced episodically as part of one's past. By contrast, the memory content psychologists classify as semantic often, though not invariably, tends to be represented as stable, summaries of (often repeated) experiences that share features in common. While such content is acted on by KC rather than by TC (i.e., autonoetic awareness), there is nothing in Dalla Barba's model that precludes KC (i.e., noetic awareness) from recruiting an individual's logical abilities to inferentially place “stable” content in a temporal context, provided the representation being addressed contains temporally-relevant constituents. When this happens, the individual is able to locate well-learned, multiply-represented facts about the world in a temporal matrix that extends from the chronological past to the chronological future (e.g., “I know I lived in New York until I was 2 years old, even though I can't recollect any specific event from that time of my life”).

Unfortunately, as the reader will have noted, Dalla Barba's “explanation” begs the question of what *causes* the observed correlation between type of temporal subjectivity and stability of content. At present, a compelling explanation is not readily at hand. Despite its conceptual limitations, however, Dalla Barba's model, in conjunction with MTT, provides a provisional (though incomplete) framework for making sense of the dissociation between the experiential and inferential forms of temporality experienced by patient R. B. As the result of a condition in which autonoetic awareness is intact, but unable to access pre-injury content in long-term memory, R. B. remains capable of describing, often in considerable detail, what happened in his pre-injury past—despite being unable to experience his present mental content as a re-living of past personal events. By contrast, autonoetic awareness still can, for reasons not clear, successfully work in conjunction with his post-injury memory content. What we see in the case of R. B. is not a failure of memory content, or a loss of the autonoetic component of recollection, but rather a dissociation between two intact, yet functionally independent, constituents of what, taken in tandem, are essential constituents of what we classify as episodic recollection.

The merit of this explanation of the relation between content and experience is further supported by R. B.'s subsequent recovery of the ability to episodically recollect the *same* memory content that lacked personal ownership during the period of his cognitive impairment. That R. B. was able to regain these functions suggests that the autoneotic aspect of his recollections was not destroyed by his injury; rather it temporarily became decoupled from memory content. The proposition that the mechanisms mediating autonoetic awareness were not lost during his amnesia also is implied by the fact that he had a sense of personal ownership of ongoing experiences that transpired *following* his accident (with the exception of temporally limited anterograde memory loss). Why his mental machinery was able to conjoin autonoetic awareness with content as memories were being built, but not when recollecting memories of pre-injury events, remains unclear.

## Conclusions

The sense of a direct, pre-reflective attachment to the past given by episodic recollection is robust, indeed, it has seemed to many to be a necessary aspect of episodic memory (for reviews, see Tulving, 1985, 2002; Wheeler et al., 1997). It also gives us an “irresistible” sense of being the same person over time (e.g., Fivush and Haden, 2003; for discussion, see Klein, 2013b). But the case of R. B. indicates that this sense is dissociable from memory content. The feeling of autonoetic personal continuity (and its intimate relation to personal ownership) turns out to be a contingent feature of memory content that comes into play at retrieval.

Seen in this light, classification of content as episodic or semantic can be situated in processes that transpire once memory content is retrieved and made available for conscious experience. It is at this point that the designators episodic and semantic do the work they were developed to perform. This work is achieved via the individual's ability to connect a current mental state to a past experience via either autonoetic awareness (temporarily lost in R. B, but subsequently regained) or logical inference. The former maps to what we call episodic memory, while the latter enables temporally located semantic knowledge—i.e., the intentional placement of retrieved content in the context of one's past (provided the content has useable temporal, spatial and self-referential markers).

Of course, R. B.'s reports do not rule out the possibility that what he is reporting are inferences based on semantic memory system (as his responses make clear, he is capable of inference) rather than “un-labeled” memory content that lacks a felt connection to his past due to compromised autonoetic awareness. However, this possibility assumes that semantic memory is a system whose instantiation is a biological reality *prior to* an act of retrieval—a conceptual stance which theoretical and empirical observations presented in this paper call into question. Moreover, Occam's principle of parsimony—i.e., posit no more parameters or variables than are minimally necessary to account for the data (e.g., Ladyman, 2002)—appears to side with the view that memory content is classifiable neither as episodic or semantic while still in storage. Of the two possibilities under discussion, the retrieval-based alternative offers the simpler explanation—positing a single source of content that can be differentially acted on by autonoetic awareness. By contrast, the traditional classification of episodic and semantic memory as unique, but interacting, neuro-cognitive systems assumes two separate repositories of content (episodic *and* semantic) which *also* are differentially associated with autonoesis.

Occam's razor resonates with evolutionary considerations as well. Evolution builds on existing biological structures (e.g., Williams, 1966). Consistent with this thesis, Fuster (1995) has demonstrated a strong overlap among humans and phylogenetically older mammalian species in the cortical areas involved in memory. Accordingly, positing a pre-existing cortical network of memory content that subsequently was overlain with mechanisms that acted differentially on retrieved content to enable conscious experience to be taken as either episodic or semantic has economically favorable consequences. Specifically, it has the effect of eliminating the need to posit the evolution of separate systems of storage for episodic and semantic content, as well as separate mechanisms (i.e., autonoetic and noetic) for consciously experiencing that content as episodic recollection or semantic knowledge.

These considerations also offer an evolutionary perspective on the well-known finding that some species (e.g., scrub jays), lacking some of the structures assumed necessary for episodic recollection (e.g., personal ownership, sense of self), nonetheless behave *as though* their acts were mediated by recollection (e.g., Cheke and Clayton, 2010). Such behavior can parsimoniously be explained by assuming these species have a network for storing memory content, some which contains information about time and place, but have yet to evolve the mechanisms necessary to place that content into subjective alignment with their personal past. Thus, they can use their knowledge to appropriately guide behavior without that knowledge being experienced as part of their personal past.

The retrieval-based model of episodic and semantic memory also may help explain the well-known finding that the *experience* of remembering often is characterized by either a feeling of knowing or a feeling of remembering (for reviews, see Cohen et al., 2008 and Gardiner and Richardson-Klavehn, 2000). A popular dual systems explanation for variation in modes of retrieval experience is that feelings of remembering reflect the operation of episodic memory whereas the feelings of familiarity are associated with semantic memory (e.g., Tulving, 1985).

In contrast to dual systems analyses, a retrieval-based model proposes that whether content retrieved from memory is experienced as remembered or as known depends on whether it has been subject to autonoetic consciousness: If it has, the content is experienced as remembered; if not, it is experienced as known. This explanation has the advantage of avoiding the problem (common to dual systems models) of explaining why stimuli encoded under the *same* temporal and spatial conditions are stored in, and subsequently retrieved from, one system vs. another. A retrieval-based model posits that all stimuli are stored in the same neural system; any difference in mode of presentation pivots on whether or not content is acted on by autonoetic awareness at retrieval.

Although this model has the advantage of conceptual and phylogenetic parsimony (e.g., a single system of storage), an obvious limitation is that identification of the factors responsible for whether retrieved content will be subject to autonoetic embellishment is, at present, unknown. However, a similar indictment can be pressed against most *process*-based explanations of the remember/known phenomenon. Regardless of whether one subscribes to a dual or single process explanation (e.g., Tulving, 1985; Gardiner and Java, 1990; Donaldson, 1996; Wixted and Mickes, 2010; for review see Cohen et al., 2008), there is no compelling a priori basis for linking variations in autonoetic awareness to the potency of causally-relevant factors (such as trace strength, fluency, decision criteria, and automaticity); rather, these processes are invoked post hoc to explain observed variation in participants' retrieval phenomenology.^5^

At present, there is no conclusive theoretical or empirical evidence to select between a systems-based and retrieval-based explanation of the episodic/semantic distinction (I address the untidy nature of neural localization studies of memory in the next section). Occam's principle, while supportive of a retrieval-based view, is best treated as a logically non-binding heuristic rather than as a definitive arbiter of theoretical substantiation. Compelling empirical evidence in support of a retrieval-based interpretation comes from the fact that, on recovery of his sense ownership, the same content formerly unconnected to R. B.'s personal past re-acquired a sense of being an episodic recollection. While definitive evidence for a retrieval-based theory of episodic and semantic memory is not available at present, R. B.'s memory performance, taken in conjunction with considerations of parsimony and evolution, suggest this option should be considered a live possibility.

### The neural localization of episodic and semantic memory

The localization of episodic (and semantic) memory in their neural substrates has been a task that has captured the interest of neuro-imagers for several decades (for reviews, see Squire, 2004; Moscovitch et al., 2006; Martinelli et al., 2012; Renoult et al., in press). However, despite guarded optimism initially expressed that the goal of localization of the relevant networks was “in sight” (e.g., Nyberg et al., 1996), the complexities underlying these early forays soon became evident. Researchers were led to conclude that the networks associated with episodic and semantic memory are widely distributed in the brain (e.g., the parietal lobes, the frontal lobes, medial temporal lobes; e.g., Nyberg and Tulving, 1998; Moscovitch et al., 2005; Wagner et al., 2005; Burianova and Grady, 2007; Squire and Bayley, 2007; Cappa, 2008; Ryan et al., 2008; Eichenbaum et al., 2012; for review, see Svoboda et al., 2006; Uttal, 2009), that their boundaries were fluid, and that their constituents varied as a function of the task performed, content retrieved, and a host of related factors such as the individual's age, handedness, gender, clinical status, and emotional state (e.g., Achim and Lepage, 2003; Bartha et al., 2003; Schwindt and Black, 2009; Smith and Squire, 2009; for review, see Dumit, 2004). Not surprisingly, even the manner in which constructs of interest were operationalized had important effects on the cortical regions activated (e.g., Renoult et al., in press). One might argue that there is as much evidence for the incoherence of the underlying constructs as there is for the complexities of the issues involved in designing studies, analyzing data and interpreting findings from brain-mapping endeavors (e.g., Uttal, 2001).

A possible reason for the diversity of imaging results is that episodic memory is not something to be neurally localized—it is not a *thing* to be found. Rather, it consists in a collection of functionally independent, but normally interacting functions (e.g., Klein, 2001; Klein et al., 2004), which, as the present study demonstrates, can differentially be impaired due to neurological damage (for reviews, see McCarthy and Warrington, 1992; Klein et al., 2004). As Polanyi (1967) cautioned “… either you know what you are looking for, and then there is no problem; or you do not know what you are looking for, and then you cannot expect to find anything” (p. 22).

Despite the difficulties of the enterprise, there are several conclusions that can provisionally be drawn from studies imaging episodic and semantic memory. Of particular relevance to present considerations, a number of labs have converged on the medial temporal lobes as a common network underlying episodic and semantic consolidation and storage (e.g., Scoville and Milner, 1957; Achim and Lepage, 2003; Bartha et al., 2003; Piolino et al., 2003; Levy et al., 2004; Moscovitch et al., 2005; Svoboda et al., 2006; Ryan et al., 2008; Smith and Squire, 2009; Naya and Suzuki, 2011; Rosenbaum et al., 2012; Eichenbaum et al., 2012). In addition, these networks also are implicated in the consolidation and storage of declarative memory content (for review, see Fuster, 1995; Squire, 2004). While highly speculative, I suggest that these regions of activation may reflect the storage of memory content prior to its subsequent demarcation as semantic and episodic via mechanisms acting at retrieval. By contrast, a number of investigators have suggested that autonoetic awareness depends on mechanisms residing primarily in the frontal lobes (e.g., Abraham et al., 2009; for reviews, see Wheeler et al., 1997; Szpunar, 2011; Tulving and Szpunar, 2012).

### The episodic/semantic dichotomy and declarative memory

An implication of my proposal, in partial agreement with Squire and his colleagues, is that cortical separation of memory systems may better be captured by a declarative/procedural dichotomy (where episodic and semantic systems are folded into, rather than constituents of, declarative long-term memory; e.g., Cohen, 1984; Squire, 2004) than by a taxonomy in which episodic and semantic memory are seen as conceptually and neurologically distinct constituents of the declarative system (e.g., Schacter and Tulving, 1994; for discussion, see Foster and Jelicic, 1999).

But—and this is an important caveat—my concession to the declarative/procedural model comes at the level of the neural instantiation of memory content, not at the level of phenomenology once that content has been retrieved and made available as an object for subjectivity. Accordingly, the episodic/semantic division of long-term memory is not subsumed by declarative memory (as Squire and colleagues might argue); rather, the episodic/semantic division of memory comes into play at the level of retrieval rather than storage.

This view can accommodate several conflicting findings in the literature. For example, while some clinical dissociations between intact and impaired memory function appear best classified in terms of a functional independence of episodic and semantic memory (for reviews, see Schacter and Tulving, 1994; Klein, 2004, 2010; Dere et al., 2008), other results do not fit as neatly into this scheme (e.g., Cohen, 1984; Squire, 1987; Kopelman et al., 1989; Klein et al., 2002c). In fact, an unambiguous sorting of spared and preserved function into the categories provided by the episodic/semantic distinction more often is the exception than the rule (e.g., Squire, 1987; Baddeley et al., 1995; Parkin, 1997; Foster and Jelicic, 1999; Klein et al., 2002c; Moscovitch et al., 2006).

However, recognition of the possibility that an episodic/semantic classification of memory impairment is attendant on contingencies acting at retrieval can accommodate the diversity of results. Specifically, impairments acting primarily on stored content may result in impairments to *both* episodic and semantic memory experience, whereas separation between these two forms of memory phenomenology is more likely to be observed when neuro-cognitive impairments act on the mechanisms operative during retrieval.

Evidence for this proposal comes from the case of patient D. B., who suffered brain damage as a result of anoxia following cardiac arrest (e.g., Klein et al., 2002b,c). Neuropsychological assessment of D. B.'s temporal orientation showed severe disorientation with respect to the present. For example, he did not know the day of the week, the current year, or even his age. Additional testing revealed that D. B. was unable to recall his past and unable to imagine what his experiences might be like in the future (for review, see Klein, 2013b). Not surprisingly, D. B.'s episodic memory was severely impaired: He could not reliably bring to mind personal experiences from any point in his past (at least within the limits of testing). By “reliably” I mean the while D. B. typically responded to requests for episodic memories with “I don't know,” he occasionally did offer a specific “recollection.” However, these “recollections” lacked rational placement in his past. For example, in response to the request that he remember a time he was in a car, D. B. replied “Driving down the coast with my parents.” When then asked to temporally place the memory, he replied “yesterday,” despite his parents having been dead for 34 years! Thus, an occurrent mental state (the content of which was verified by his daughter) appears to have broken free of its temporal moorings. That is, the content of memory, absent autonoetic temporal placement, constituted the object of D. B.'s awareness.

What I am arguing is that patients such as R. B. and D. B. may suffer from a disruption of autonoetic awareness, and that as a result of this pathology they are rendered unable to experience mental content in its proper temporal context (for a similar views, see Tulving, 1985; Dalla Barba, 2002). It is the failure to connect autonoetic awareness with retrieved content rather than the absence of such awareness, that accounts for (at least some) of the memory pathologies demonstrated by amnesic patients.

This is not to presume that all forms of episodic amnesia submit to similar analysis. Memory loss can result from failures at encoding, storage and/or retrieval. One could, for example, present symptoms consistent with episodic amnesia if s/he maintained the requisite mechanisms for temporal subjectivity, but lacked access to the content on which that awareness could be brought to bear. Content loss can be highly selective. According to MTT, less stable (and hence more autonoetically-relevant) content is likely to be under-represented in memory. It thus is more susceptible to neural insult than are the more richly distributed representations that ultimately will constitute semantic memory experience (e.g., Nadel and Moscovitch, 1997). In this way, episodic amnesia will manifest more readily than sematic amnesia (as is well-recognized to be the case)—the result of disease processes acting on content storage prior to operations taking place at retrieval. What I am suggesting, then, is that some (though certainly not all!) forms of amnesia can arise from the decoupling of temporal awareness and memory content (for a similar view, see Tulving, 1985).

Another area of research that submits to a retrieval-based analysis is the debate surrounding the mechanisms underlying false memories (for review, see Schacter, 1995; Johnson and Raye, 1998). According to the present model, memory errors can result when (1) compromised content is taken as the object of autonoteic awareness as well as (2) when autonoetic awareness is misapplied (e.g., to imagination). The phenomenon of implicit task performance also can be explained (in some cases) as the failure of autonoetic awareness to place memory content in a temporal context. While both of these phenomena (false and implicit memory) deserve considerably more attention, restrictions of space prevent further elaboration.

### Limitations and considerations

An issue raised concerning the model I have presented is that it seems more a philosophical exercise that a scientific theory. Concern centers on the fact that evidence in support of my ideas derives primarily (though not exclusively: e.g., patient D. B, thought insertion) from the study of a single patient (R. B.) who now has remitted. Accordingly, it is hard to see how the ideas I have proposed can be tested via experimental manipulation. There are several things to note in this regard.

First, not all theory-based scientific enterprises admit to empirical *manipulation* (e.g., cosmology, paleontology). While the potential for refutation is essential (e.g., Popper), refutation does not mandate explicit manipulation in an experimental context (e.g., Trusted, 1987; Lipton, 1991). A good theory is one that retrodicts and predicts, both of which afford the potential for refutation in the absence of the ability to directly manipulate variables of interest.

Second, a good theory facilitates the organization of data sets that might otherwise have been viewed as collections of unrelated findings (e.g., Newell, 1973; Ladyman, 2002; Godfrey-Smith, 2003). Along these lines, the present theory has the virtue of offering a parsimonious explanation for a variety of “apparently” diverse memory phenomena, including, but not limited to, remember/know judgments, the implicit/explicit memory distinction, false memories and Déjà vu experience (to be discussed in a forthcoming paper). It also can account for the fact that some individuals suffering Dissociative Identity Disorders experience the *same* episodic content (e.g., Dorahy, 2001) despite content ownership varying as a function of the personality currently occupying awareness (e.g., Braude, 1995). The theory also accounts for the fact that episodic and semantic content evidence significant (often indistinguishable) overlap with regard the presumably differentiating features of time, place and self (e.g., Klein and Lax, 2010).

Third, the availability of theory can help to draw attention to neurological case studies whose relevance for the study (in this case, of memory) has, to date, been underappreciated. For example, Zahn et al. (2007) report the case of a patient D. P., who lost his ability experience ownership of the mental states accompanying perceived objects. In a case described by Gott et al. (1984), the patient (J. J.) was capable of holding either of two qualitatively different states of consciousness. Specifically, she was voluntarily able to switch between the feeling that her experiences belonged to her or to someone else. Finally, a patient described by Sass and Parnas (2003) reported that his feeling ownership accompanying his mental states lagged behind his initial awareness of those states. This “phenomenological delay in felt ownership” suggests that ownership is a separable component of consciousness—i.e., the patient temporarily was aware of having an experience, but only subsequently felt that his first-person perspective belonged to him.

Taken together, these studies focus attention on ownership as an aspect, or form, of consciousness that can come undone under certain conditions (e.g., Klein and Nichols, 2012; Lane, 2012). With respect to the theory of episodic memory I have proposed, these studies potentially constitute a small data-base (I suspect additional cases will appear in the literature if dysfunction of content ownership becomes a recognized issue in memory research) that, once assembled, will permit investigators to empirically test the effects of “loss of ownership” on memorial experience.

Fourth, as things currently stand, my theory also submits to empirical testing with *non*-impaired participants. Here I outline one such study (others will be discussed in a forthcoming paper). Assuming it possible to *simultaneously* achieve a high degree of temporal and spatial resolution using various brain scanning technologies (e.g., EEG, fMRI, and PET) it should be feasible to track both the chronology and localization of neural activity during performance of a “remember/know” task. If temporal resolution is sufficiently sensitive, and the constructs in play sufficiently well defined (note: “sufficiently” may require technological and conceptual refinement), it would allow us to examine the stages of memory (i.e., encoding, storage, and retrieval) activated during the process of declarative remembering. Anatomical localization—in conjunction with “stage” information provided by temporal data—would enable us to bring to focus the systems responsible for storage (which are, of logical necessity, causally prior to retrieval) followed by those involved in retrieval. If my model has merit, the activation of stored content during performance of the remember/know task will evidence comparable localization(s) regardless of whether the participant's phenomenological report turns out to be “remember” or “know.” Neural separation, by contrast, should be evidenced during the retrieval phase (due to differences in the mechanisms mediating remember/know judgments—i.e., inference vs. autonoetic awareness).

Finally, the notion that single case studies have serious limitations with regard to theory construction is far from agreed on. In fact, Caramazza (1986, 1991) and Sokol et al. (1991) have argued persuasively for the importance of *N* = 1 studies in the development of neuropsychological models. Of course, not everyone shares this view: Some feel that theory construction requires inference from group performance (e.g., Shallice, 1988; Robertson et al., 1993). However, since there are no “knockdown” arguments favoring one view to the exclusion of the other, there is, at present, no logically compelling reason for conceptual closure.

The theory presented herein also offers a potential corrective on research practices that may be doing more to cloud than to illuminate the role of long-term memory in various task performances. A central idea of this paper is the episodic and semantic memories are distinguished not by their content, but rather by the way that content is phenomenologically given: The features of “episodic content” are not, in principle, distinguishable from those of “semantic content.” This calls into serious question the advisability of studies attempting to document the workings of a particular type of declarative memory via analysis of reported remembered content. To take one example (and there are a multitude), a recent paper by Rasmussen and Bernsten (2012) attempts to document the episodic contributions to future-oriented thought by examining the relative proportions of episodic and semantic content present in participants' memory transcripts. Such an attempt, on present considerations, is misguided since there is no principled way in which a researcher can classify content as episodic or semantic; the episodic and semantic designationors refer to the manner in which content is experienced at retrieval.

### Final thoughts

The present emendation of the episodic/semantic memory distinction awaits the considerable work of empirical conformation and theoretical accommodation. However, despite its provisional status, it has the merit of (1) being consistent with real-world data from case studies (e.g., R. B., and D. B.), (2) helping bring some order to the debate over whether episodic and semantic memory are best construed as functionally independent neural systems, or rather two ways scientists (though not necessarily nature) have chosen to divide up declarative memory, (3) helping make sense of impairments in mental time travel (both into the past and future; for discussion, see Klein, 2013a), (4) accommodating a number of findings (e.g., the differential bases for, and forms of, episodic amnesia, memory errors, and performance on implicit memory tasks), and (5) having considerations of parsimony (both logical and evolutionary) on its side.

The decoupling of autonoetic awareness and memory content—revealed most clearly by the case of R. B. (see also the case of patient D. B)—can be taken as an “existence proof” for the proposition that the connection between autonoetic awareness and episodic memory is one of contingency rather than one of necessity. That is, R. B.'s memory phenomenology suggests that awareness is not an *intrinsic* property of episodic content; rather, the association between content and awareness may best be construed as a *relation* between two functionally independent systems that jointly contribute to the experience of episodic recollection.

Perhaps ironically, Hermann Ebbinghaus, a name typically associated with an approach to memory now discredited as being overly-influenced by logical positivism (e.g., Bartlett, 1932; Danziger, 2008), seems to have intuited (though not explored) the need to invoke acts of consciousness to explain memory phenomenology. In the introductory remarks in his book *Memory*, Ebbinghaus makes the following observation: “… in the majority of cases we at once recognize the returned mental state as one that has already been experienced; that is, we remember it. Under certain conditions, however, this *accompanying* consciousness is lacking and we know only indirectly that ‘now’ must be identical with ‘then’… ” (Ebbinghaus, 1885/1913, p. 2; emphasis mine). Put in terms of the model proposed in the present paper, Ebbinghaus is calling attention to the need to posit an autonoetic-like form of consciousness that acts on retrieved memory content to provide the experience of pastness as directly given (i.e., episodic) rather than indirectly inferred (i.e., semantic).

### Conflict of interest statement

The author declares that the research was conducted in the absence of any commercial or financial relationships that could be construed as a potential conflict of interest.

## Footnotes

^1^Although James also is clear that much of what we now call semantic knowledge would not qualify as memory in his system—lacking, as it does, obvious reference to one's past (e.g., 2 + 2 = 4; the sun is hot)—I believe that reference to the past is overly restrictive and not a necessary criterion for a mental experience or bodily action to meet the requirements for being a memory. A memory is an occurrent mental event that can be shown to have a causal connection to past experience. On this view, the expression of learning (whether via mental state or physical act) qualifies as memorial regardless of whether its current instantiation can be consciously traced to, or felt to derive from, a previously encountered event or experience, provided the proper causal connection(s) to a past experience can be demonstrated (via inference, objective evidence, or felt experience of past). For example, intelligently coordinated movements of the body which draw on the accumulated effect of knowable past events qualify as memorial despite absence of awareness that they are causally derived from previous experience (i.e., procedural memory). Of course, a little thought reveals an obvious circularity in any criterion of memory that draws on acquaintance (direct or indirect) with the past (i.e., memory) as the evidential basis for memory (e.g., Furlong, 1951). This perplexing conundrum would take us too far from the issues at hand and thus will not be dealt with here.

^2^One limitation of the present account is that I have no proven criteria for specifying when autonoetic awareness will act on retrieved content other than the trivially obvious consideration of content relevance to temporal treatment [One speculative possibility—which I develop later in this paper—trades on Nadel and Moscovitch's (1997) Mutliple Trace Theory]. However, while the lack of a clear understanding of how and when autonoetic awareness makes contact with current mental content is an obvious weakness of my proposal, I do not think the absence of research on a previously un-proposed emendation to a well-established model argues, by itself, against the potential worth of my suggested revision. Rather, it highlights the novelty of the proposal and the need for conceptual and empirical work before the ideas expressed herein can transition from plausible to likely.

^3^It is important to note that ownership of one's mental states admits to several instantiations. For example one can have “perspectival” ownership of the content of a mental state. This implies only that one is aware that the experienced state is taking place in his or her mind. R. B. clearly maintains this form of ownership. By contrast, “personal” ownership is lacking in R. B. While he understands that the memories he experiences present themselves to his consciousness, he does not feel as though they are his own. Lacking this form of ownership, he is unable to autonoetically experience the content of his mental states as expressly referring to events from his past. For detailed discussions of forms of ownership, see Albahari (2006) and Locke (1968).

^4^Note that despite his ownership/autonetic limitations, R. B.'s responses exhibit a clear sense of self both narratively and factually. Thus, it is reasonable to conclude that his memory issues are not traceable to impairments of self (Klein et al's, 2004, subsystem #1). In addition, throughout his period of impairment he exhibits a clear and precise ability to perform a requested retrieval, thus demonstrating intact agency with regard to memory (which suggests, in turn, that subsystem #2 has not unduly been impacted by his injuries). That he has an intact sense of agency also can be seen from his remarks as the interview continues.

^5^To quote Bartlett (1932), the processes invoked “… may show us what can happen when recognition takes place, but throw no light whatever upon how any, or all, of these processes are rendered possible … experimenters have analyzed the final stage of recognition and each has tended to claim a complete solution in terms of his particular analysis. In fact, nobody can understand recognition by confining his attention to what happens at the moment of recognition.” (p. 192). Bartlett goes on to say that future experimentation focusing on the mental events that precede recognition will aid in understanding variability in the processes subsequently at work. If one substitutes retrieval (e.g., recall and recognition) for recognition, the quote from Bartlett perfectly captures my critique of process-based explanations of the remember/know phenomenon. This critique applies to my retrieval-based proposal as well: As it currently stands, a retrieval-based model cannot account for variability in the attachment of autonoetic awareness to memory content. This is not cause for dismay; rather, as Bartlett notes, it simply points out the need for further experimentation designed to clarify how the processing differences apparent at retrieval are put into place.
